# Comparative Evaluation of Flexural Toughness of Steel Fiber-Reinforced Concrete Beams

**DOI:** 10.3390/ma16103789

**Published:** 2023-05-17

**Authors:** Hyun-Do Yun, Ki-Bong Choi, Won-Chang Choi

**Affiliations:** 1 Department of Architectural Engineering, Chungnam National University, Daejeon 34134, Republic of Korea; wiseroad@cnu.ac.kr; 2 Department of Architectural Engineering, Gachon University, Seongnam-si 13120, Republic of Korea; kbchoi@gachon.ac.kr

**Keywords:** steel fiber-reinforced concrete (SFRC), tensile strength, flexural toughness, energy absorption capacity, residual strength

## Abstract

Specifications are available to quantify flexural performance of steel-fiber reinforced concrete beams with several parameters. Each specification provides different results. This study comparatively evaluates existing flexural beam test standards that are used to evaluate the flexural toughness of SFRC beam specimens. Two standards, EN-14651 and ASTM C1609, were followed to test SFRC beams under the three-point bending test (3PBT) and the four-point bending test (4PBT), respectively. Both normal tensile strength steel fiber (1200 MPa) and high tensile strength steel fiber (1500 MPa) in high-strength concrete were considered in this study. The reference parameters recommended in the two standards, which include equivalent flexural strength, residual strength, energy absorption capacity, and flexural toughness, were compared based on the tensile strength (normal or high) of the steel fiber in high-strength concrete. The 3PBT and 4PBT results indicate that both standard test methods yield similar results to quantify the flexural performance of SFRC specimens. However, unintended failure modes were observed for both standard test methods. The adopted correlation model shows that the flexural performance of SFRC is similar for 3PBTs and 4PBTs, but the residual strength obtained from the 3PBTs tends to be greater than that obtained from 4PBTs with an increase in the tensile strength of steel fiber.

## 1. Introduction

The inclusion of steel fiber in a concrete mixture can improve the flexural resistance and toughness of the concrete. In particular, flexural toughness is considerably increased in high-strength concrete mixtures [1,2]. To evaluate the flexural behavior of steel fiber-reinforced concrete (SFRC), researchers have analyzed both the toughness and energy absorption capacity of SFRC specimens. The flexural performance of SFRC can be evaluated differently depending on the evaluation method. Current specification standards in American and European standard specifications propose the three-point bending test (3PBT) and four-point bending test (4PBT) for the evaluation of SFRC performance. Alternatively, a round panel test is available, but the 3PBT and 4PBT are the most common tests used to assess the flexural performance of SFRC.

The 3PBT, which induces cracks by forming a notch at the midpoint of the specimen, is recommended in EN-14651 [3] and RILEM TC-162 [4]. The 4PBT uses deflection measurements and is recommended in ASTM C1609 [5]. Unlike the ASTM C1609 standard that is used to measure deflections in the 4PBT, EN-14651 and RILEM TC-162 are applied to measure the crack mouth opening displacement (CMOD) in the 3PBT to evaluate the relationship between flexural strength and CMOD values.

In addition to a single-notch crack, multiple cracks around the notched area can also be induced, which causes the distribution of stress in 3PBT. Accordingly, the single-notch 3PBT might not adequately represent the flexural behavior of an SFRC specimen. To consider multiple cracking patterns, ASTM C1550-10 [6] proposes the round panel test method, which some researchers prefer over bending tests to understand the flexural behavior of SFRC specimens [7].

In the 4PBT, which follows ASTM C1609, the flexural stress versus deflection relationship is evaluated by measuring the deflection of the specimen at its midpoint. However, 4PBT results are significantly impacted by the specimen geometry and test set-up due to the cross-section of the specimen and settlement conditions at the supports, which can lead to substantial scatter in the test results.

Specifically, the degree of restraint at the supports affects the flexural response and crack development in the specimen [8]. The 4PBT recommended in ASTM C1609 is intended to induce cracks at the constant moment region. However, cracks often occur at unintended locations, mostly at the loading points, because the specimen is subjected to both flexure and shear [9]. ASTM C1069 states that the test results cannot be used when failure occurs outside the pure bending region. Many studies have been conducted to overcome the limits of the existing specifications to determine flexural performance [10,11,12].

Conforti et al. proposed the use of normalized displacement using crack tip opening displacement (CTOD) to determine the correlation between the residual strength in the 3PBT and the 4PBT [13]. The test variables used were different dosage values, tensile strength values, and type of fiber. Conforti et al. found that flexural tensile strength is directly proportional to crack deformation, regardless of which flexural test is performed [13].

Galeote et al. attempted to develop a model to discern the correlation between the 3PBT and the Barcelona test of SFRC [14]. They concluded that a correlation between the two tests is not possible due to the differences in crack formation in the two tests. Li et al. pointed out that the conventional specifications are not suitable for the specimens having higher deflection to evaluate the flexural toughness [15].

Carrillo et al. [16] adopted the correlation model proposed by Conforti et al. [15] and used CTOD values obtained from the 4PBT and the 3PBT. The test variables used in the Carrillo et al. study were dosage (20 kg/m^3^, 40 kg/m^3^, and 60 kg/m^3^), geometry of hooked ends, and the tensile strength of the fiber (f_t_ = 1160 MPa, 1500 MPa, and 2300 MPa) [16]. However, the Carrillo et al. tests were conducted using 24 MPa as the compressive strength of the concrete, which is a primary parameter that is used to determine the flexural performance of SFRC [16]. Wang et al. investigated the flexural toughness of ultra-high performance concrete with variables of the steel fiber content and concrete strength [17]. They concluded that the relationship between crack mouth opening displacement and mid-span deflection in RILEM TC162 is only suitable for normal steel fiber-reinforced concrete.

In this study, the high compressive strength of concrete of 70 MPa was considered in order to extend the adopted correlation model proposed by Carrillo et al. [16]. The effects of the high compressive strength of the concrete on the tensile strength of the steel fiber were also considered. The extensive experimental results found in the literature were used in this study to investigate the comparative reliability of EN-14651 (for 3PBTs) and ASTM C1609 (for 4PBTs) to evaluate the flexural behavior of SFRC in terms of equivalent flexural strength, residual strength, flexural toughness, and energy absorption capacity.

## 2. Experimental Program

### 2.1. Materials and Specimen Preparation

Ordinary Portland cement and silica fume were mixed to achieve a concrete mixture with the target concrete compressive strength of 70 MPa. This concrete mixture contained less than 5 mm of fine aggregate and less than 13 mm of coarse aggregate. Table 1 shows the mix proportions of the resultant SFRC. The water-to-binder (W/B) ratio was 33% and the steel fiber dosage of 58.9 kg/m^3^ was in accordance with the minimum dosage of 60 kg/m^3^ recommended in American Concrete Institute (ACI) 318-19 [18] for the steel fiber replacement of steel reinforcement in shear mode.

**Figure 1:**
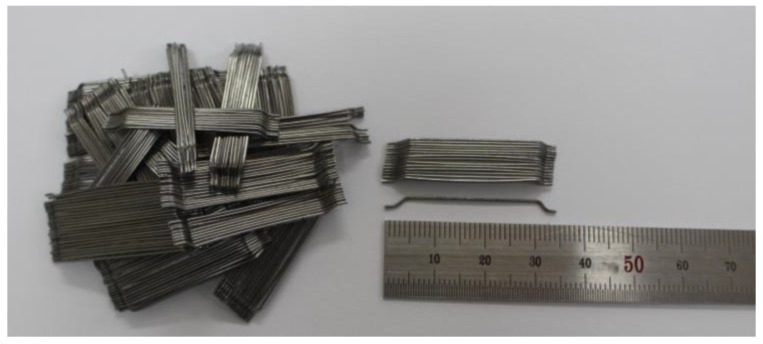
Shape and length (cm) of hooked-end steel fiber.

The structural behavior of the SFRC beam specimens was evaluated using both normal-strength and high-strength steel fiber. The type of steel fiber is hooked-end, which was reported by Soroushian and Bayasi [19] to provide the best performance over other types of steel fiber. Figure 1 shows the shape and length of the hooked-end steel fiber used in this study, and Table 2 provides a summary of its characteristics, including the mechanical properties and aspect ratio of 64 for both the normal-strength and high strength steel fiber and the tensile strength values of 1200 MPa and 1600 MPa, respectively.

### 2.2. Test Set-Ups

**Figure 2:**
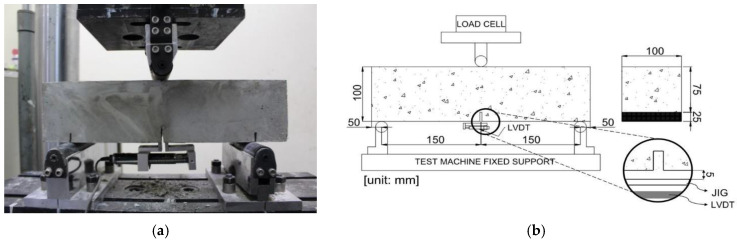
Flexural three-point bending test set-up: (**a**) test device and (**b**) EN-14651 schematic diagram showing dimension details.

**Figure 3:**
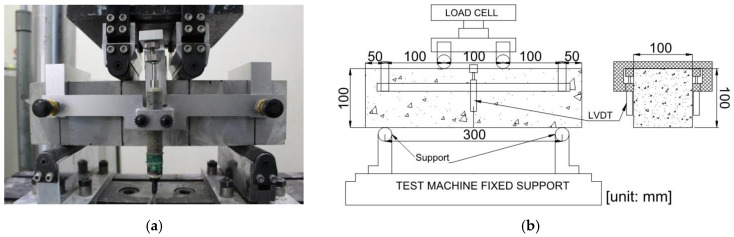
Flexural four-point bending test set-up: (**a**) test device and (**b**) ASTM C1609 schematic diagram showing dimension details.

The compressive strength of the SFRC was determined in accordance with ASTM C39 [20]. The compression behavior of the SFRC was evaluated via load control mode (0.5 MPa/s) using a universal testing machine (UTM) with 2000 kN capacity. The compression strain was measured using a compressor meter. The flexural strength of the SFRC beam specimens was determined via 3PBTs and 4PBTs. For both types of flexural tests, a UTM with a capacity of 200 kN was used with a loading rate of 0.2 mm/min. Figure 2 and Figure 3 show the standard flexural test set-ups for the 3PBT and 4PBT, respectively.

The 3PBT is a test method recommended in EN-14651 and RILEM TC-162. The geometry of a 3PBT specimen is 100 mm × 100 mm × 400 mm with a single notch cut at the midpoint of the specimen. In this study, the single notch was generated the day before testing. A steel attachment was placed to measure the CMOD at the middle of the specimen. The failure mode was recorded at the specified CMOD values of 0.5 mm, 1.5 mm, 2.5 mm, 3.5 mm, and at total failure.

The 4PBTs were performed in accordance with ASTM C1609. The 4PBT specimen has cross-section dimensions of 100 mm × 100 mm with a span length of 400 mm. The vertical displacements were recorded at each interval during the test.

## 3. Experimental Results

### 3.1. Compressive Strength Tests

**Figure 4:**
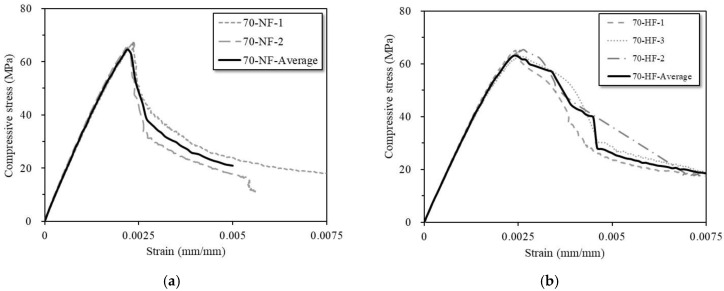
Compressive stress versus strain curves for specimens with (**a**) normal-strength steel fiber (70-NF) and (**b**) high-strength steel fiber (70-HF).

Figure 4a,b show the stress versus strain curves for the SFRC specimens with normal-strength and high-strength steel fiber, respectively. Note that 70-NF and 70-HF designate specimens with 70 MPa as the SFRC compressive strength and normal-strength steel fiber (NF) or high-strength steel fiber (HF). Figure 4a indicates that the specimens with normal-strength steel fiber exhibited brittle failure and that the SFRC compressive strength dropped abruptly after reaching peak strength. Figure 4b shows that the specimens with high-strength steel fiber failed more progressively. In short, the crack propagation and post-peak behavior of the cylindrical SFRC specimens were affected by the tensile strength of the steel fiber. During the tests, no spalling was observed in any specimens due to the effects of the steel fiber at the surface of the concrete cylinder.

Table 3 provides a summary of the measured compressive strength values obtained from all the tests. The literature indicates that the inclusion of steel fiber somewhat improves the compressive strength of concrete (*f_cu_*) because the fibers serve to prevent crack propagation and crack openings. In this study, the average compressive strength values were 67.2 MPa and 64.5 MPa for the SFRC cylindrical specimens with normal-strength and high-strength steel fiber, respectively, thus indicating an insignificant difference in compressive strength between the two types of SFRC specimens. The modulus of elasticity (*E_c_*) values are also similar: 33.6 GPa and 31.3 GPa for the normal-strength and high-strength specimens, respectively. The results show that the tensile strength of embedded steel fiber does not meaningfully affect the compressive strength of SFRC.

### 3.2. Flexural Behavior Tests

#### 3.2.1. Load versus Deflection Curves

**Figure 5:**
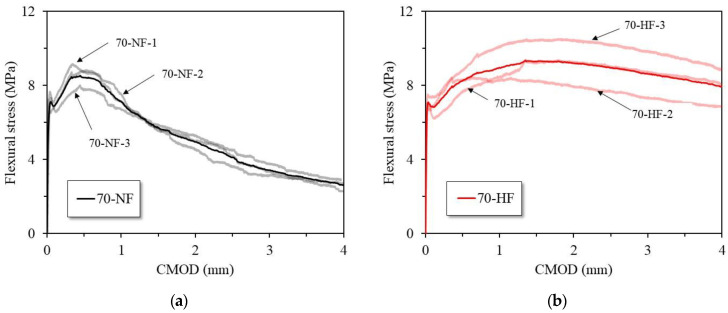
Flexural stress versus crack mouth opening displacement (CMOD) test results: SFRC specimens with (**a**) normal-strength steel fiber and (**b**) high-strength steel fiber.

Figure 5a,b show the flexural stress versus CMOD curves in accordance with EN-14651 (3PBT) for the specimens with normal-strength fiber and high-strength fiber, respectively, and Table 4 provides a summary of the effects of the tensile strength of the fiber on the flexural behavior of the SFRC. The highest flexural strength in the interval of 0.05 mm is similar at 7.43 MPa and 7.19 MPa regardless of the fiber’s tensile strength (normal or high). The maximum average flexural strength values are 8.68 MPa and 9.46 MPa, respectively. Also, as the fiber tensile strength increases, the flexural strength also increases. Based on the evaluation of the SFRC specimens’ flexural behavior, the deformation-hardening characteristics of both composites show that as the load increases, the CMOD increases, even after the initial crack. However, a significant difference in the flexural behavior is evident after 0.5 mm of CMOD. The use of high-strength steel fiber is shown to yield excellent flexural behavior of the SFRC.

**Figure 6:**
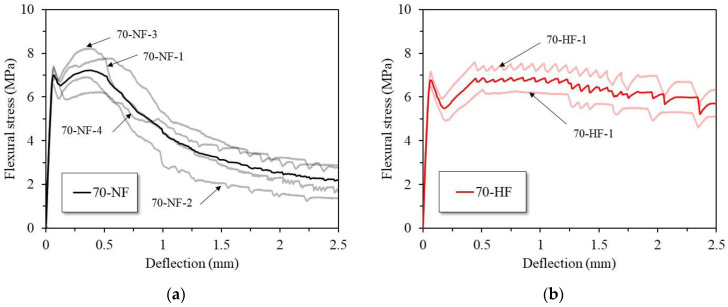
Load versus deflection curves: (**a**) specimens with normal-strength steel fiber and (**b**) specimens with high-strength steel fiber.

Figure 6a,b show the load versus deflection curves for the SFRC specimens with normal-strength steel fiber and high-strength steel fiber, respectively, and Table 5 presents a summary of the flexural test results in accordance with ASTM C1609 (4PBT). The first peak strength values of the SFRC specimens, which is due to the resistance of the contribution of the concrete, are similar at 7.00 MPa and 6.86 MPa for the specimens with normal-strength and high-strength steel fiber, respectively, and the first-peak deflections are similar at 0.06 mm. The peak strength of the specimen with normal-strength fiber is 7.56 MPa and the specimen with high-strength fiber shows 7.05 MPa of flexural strength. The net deflections at the peak strength are 0.32 mm and 0.25 mm for the specimens with normal-strength and high-strength steel fiber, respectively. The test results for the specimens with normal-strength fiber indicate deflection-hardening behavior after the development of initial cracks. However, the load decreased rapidly after the net deflection of the peak load. For the specimens with high-strength steel fiber, the deflection-hardening behavior occurred after the peak load and continued to the end of the test. The specimens with high-strength steel fiber showed more ductile behavior compared to the specimens with normal-strength fiber.

Based on the load versus deflection curves, the residual strength was computed at the net deflections of 1/600 and 1/150 of the span length. The equivalent flexural strength, f_e,150_, was computed using Equation (1) and then the equivalent flexural strength ratio (R_150_) was computed using Equation (2).

(1) $$f_{e,150}^{D}=\frac{150\cdot T_{150}^{D}}{b\cdot d^{2}}$$

(2) $$R_{T,150}^{D}=\frac{f_{e,\;150}^{D}}{f_{1\;}}\cdot 100\%$$

where *T*_150_ is the area under the load versus net deflection curve (Joule) and *f*_1_ is the first peak strength (MPa).

#### 3.2.2. Failure Modes of SFRC Beam

**Figure 7:**
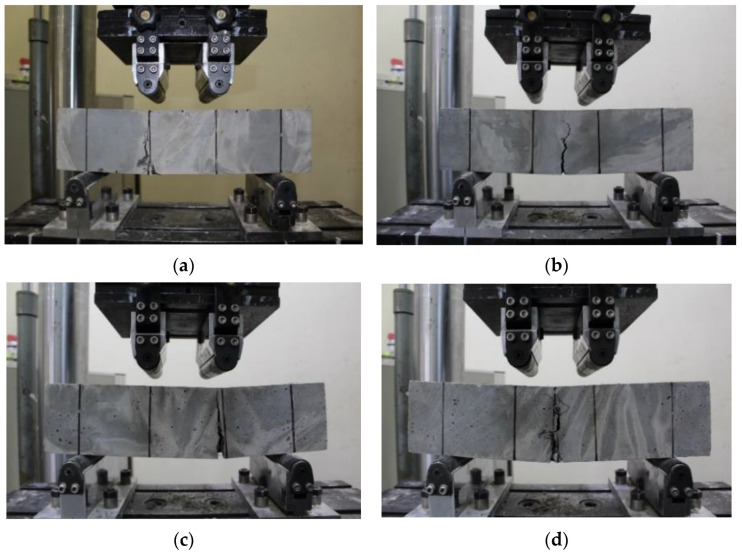
Failure modes of steel fiber-reinforced concrete specimens under ASTM C1609 four-point bending tests: (**a**) specimens with normal-strength steel fiber with unintended crack, (**b**) specimens with normal-strength steel fiber with intended crack, (**c**) specimens with high-strength steel fiber with unintended crack, and (**d**) specimens with high-strength steel fiber with intended crack.

The failure mode of the steel fiber was also analyzed in this study because the difference in flexural performance according to the fiber’s tensile strength (normal or high) is related to the failure mode of the steel fiber. Figure 7 shows the failure modes of the normal-strength and high-strength steel fiber with intended cracks and unintended cracks according to ASTM C1609 standards for 4PBTs. The 4PBT is designed such that cracking occurs at the constant moment region, as shown in Figure 7b,d for specimens with normal-strength and high-strength fiber, respectively, and intended cracks. Cracks often occur at an unintended crack location mostly at the loading points, as shown in Figure 7a,b for specimens with normal-strength and high-strength fiber, respectively, and unintended cracks, because the specimen is subjected to flexure and shear. Regardless of the tensile strength of the steel fiber, unintended cracking was observed in this study.

**Figure 8:**
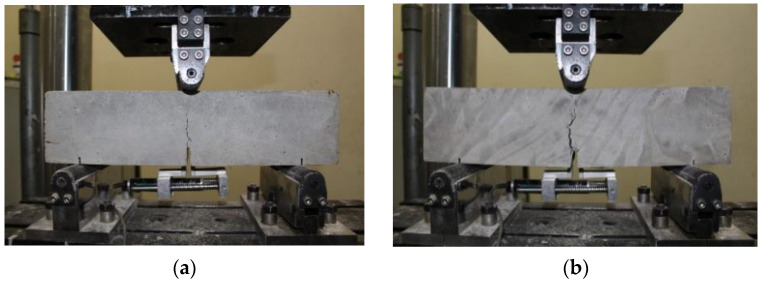
Failure modes of steel fiber-reinforced concrete specimen under EN-14651 three-point bending tests: (**a**) intended single-notched cracking and (**b**) unintended multiple cracking.

Figure 8 shows the failure modes of the SFRC specimens according to EN-14651 standards for 3PBTs. Figure 8a shows the intended cracking that initiated around the single-notched region. Figure 8b shows the unintended multiple cracking that developed and propagated around the single crack.

Regardless of the tensile strength of the steel fiber, unintended cracking was observed in this study for both 3PBTs and 4PBTs.

## 4. Discussion

### 4.1. Energy Absorption Capacity

**Figure 9:**
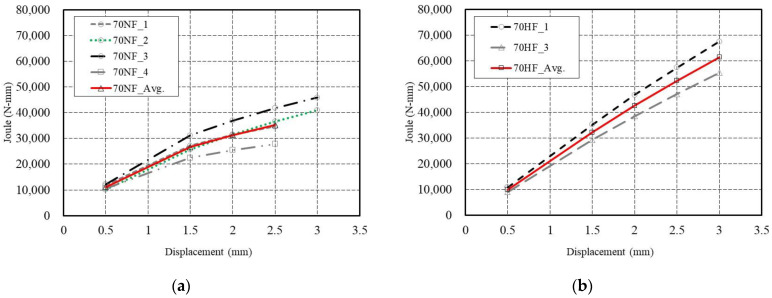
Energy absorption capacity in ASTM C1609 standard for 4PBTs: (**a**) specimens with normal-strength steel fiber (70-NF) and (**b**) specimens with high-strength steel fiber (70-HF).

The energy absorption capacity of SFRC is associated with the flexural toughness of SFRC beams, which is represented by the area of the load versus deflection curve up to a certain deflection point. As per ASTM C1609 standards for 4PBTs, two deflection points of L/600 (0.5 mm) and L/150 (2.0 mm) were determined in this study. The standard test method was extended to include deflection points of L/300 (1 mm), L/200 (1.5 mm), and L/120 (2.5 mm) to consider the excellent load carrying capacity after reaching the peak load. Figure 9 shows the energy absorption capacity of SFRC specimens at the specified deflection points. The results clearly indicate that the energy absorption capacity from the peak load to the end-point of the specimens with high-strength steel fiber is greater than that of the specimens with normal-strength steel fiber. The initial energy absorption capacity up to the peak load is similar, regardless of the tensile strength (normal or high) of the steel fiber.

The EN-14651 standard for 3PBTs addresses the correlation between CMOD and vertical deflection (δ) in Equation (3). This relationship is applicable in the post-peak region of the load versus CMOD curve.

CMOD = 1.18 δ + β(3)

where δ is vertical deflection at mid-point and β = −0.0416 mm.

The energy absorption capacity (D_BZ,2_ and D_BZ,3_) is the area under the load versus deflection curve up to the deflections of δ_2_ and δ_3_, where δ_2_ and δ_3_ are determined as the deflections at the limit of proportionality δ_L_ plus 0.65 mm and 2.65 mm, respectively.

**Figure 10:**
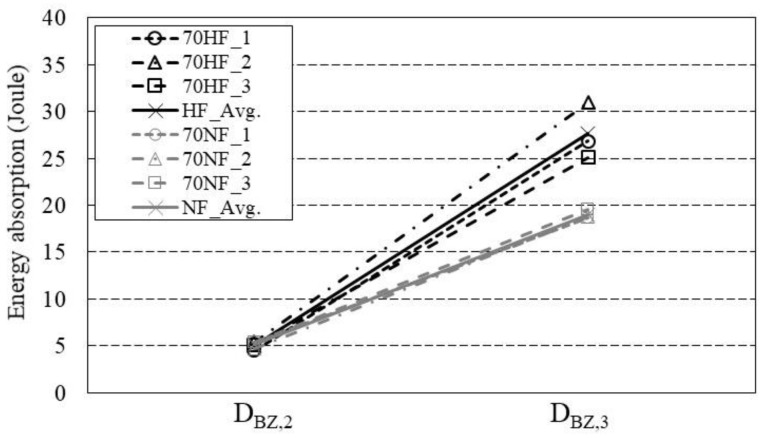
Energy absorption capacity as described in RILEM TC 162-TDF.

Figure 10 indicates that the energy absorption capacity D_BZ,2_ is similar for all specimens regardless of the tensile strength of the steel fiber. D_BZ,3_ increases with an increase in the tensile strength of the steel fiber. Specifically, D_BZ,3_ of the specimens with high-strength tensile strength fiber is 46% greater than that of the specimens with normal-strength steel fiber.

In short, both standards, ASTM C1609 and RILEM TC 162-TDF for 4PBTs and 3PBTs, respectively, similarly compute the resistance as the contribution of the concrete. The energy absorption capacity recommended in both standards clearly indicates that the tensile strength of the steel fiber affects the residual strength of SFRC.

### 4.2. Effect of Steel Fiber Volume Fraction

**Figure 11:**
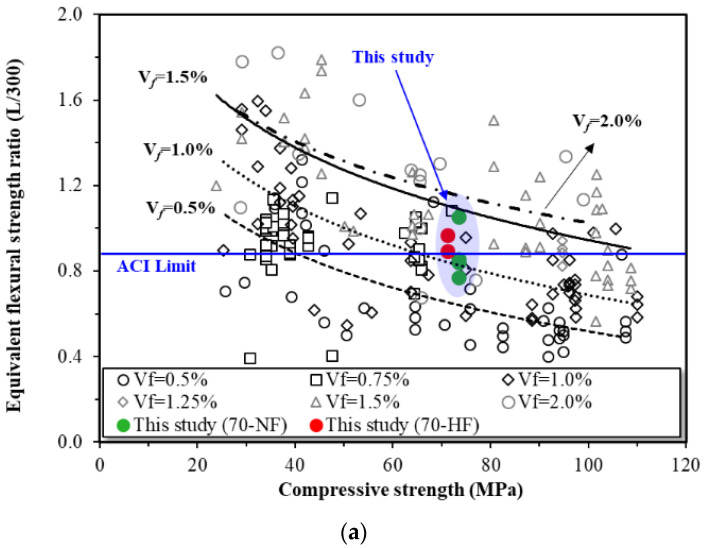

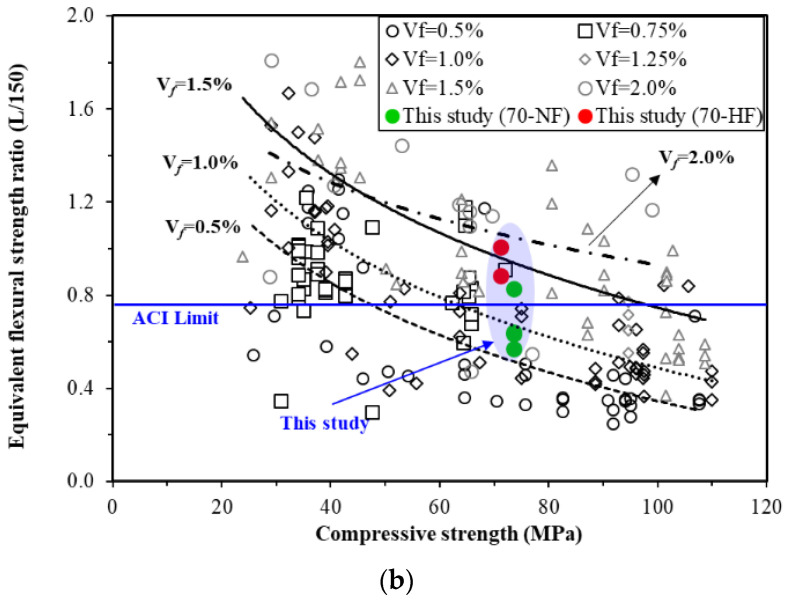
Effects of fiber strength on equivalent flexural strength ratio of SFRC: (**a**) deflection of L/300 and (**b**) deflection of L/150.

Figure 11a,b show the equivalent flexural strength ratios of the specimens at the specified deflections of L/300 and L/150, respectively. The equivalent flexural strength ratio is computed using Equations (1) and (2) in accordance with ASTM C1609. Figure 11 also presents test results obtained from other studies [21,22,23,24,25,26,27,28,29].

ACI 318-19 states that steel fiber can replace minimum shear reinforcement when the residual stress at the deflection of L/300 is more than 90% and the residual stress at the deflection of L/150 is over 75 percent. In this study, for the equivalent flexural strength ratio at deflections of L/300 and L150, some specimens with normal-strength steel fiber did not satisfy the ACI criterion. However, the standard was barely satisfied for the specimens with high tensile strength steel fiber. The equivalent flexural strength seems to be affected by the compressive strength of the concrete and the volume fraction of the fiber. Under the same compressive SFRC strength, the high tensile strength of the steel fiber tends to correspond to an increase in the equivalent flexural strength ratio.

**Figure 12:**
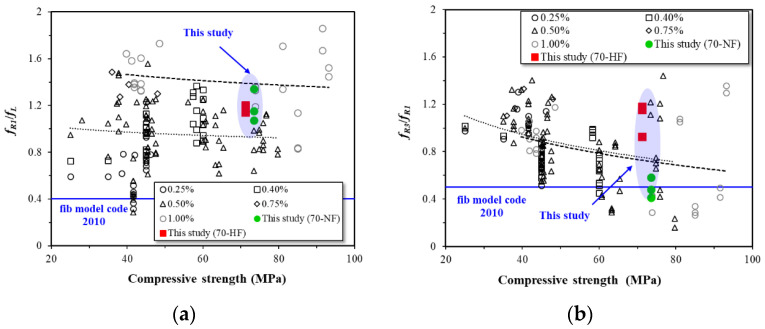
Effects of fiber strength on residual strength ratio of SFRC: (**a**) ratio of *f_R_*_1_ and *f_L_* and (**b**) ratio of *f_R_*_3_ and *f_R_*_1_.

Similarly, the residual strength ratio was computed according to the International Federation for Structural Concrete (fib) Model Code 2010 [30]. Figure 12a,b show the residual strength ratios at the specified CMOD values. The fib Model Code 2010 indicates that steel fiber may replace conventional reinforcing bars if certain criteria are satisfied. In this study, the residual strength ratios at CMODs of 0.05 mm and 0.5 mm are 1.19 and 1.18, respectively, which exceed the 0.4 limit values in fib Model Code 2010. However, for the second criterion (f_R3_/f_R1_), the residual strength ratios at CMODs of 0.5 mm and 2.5 mm are 0.49 and 1.09, respectively, which shows that SFRC with normal-strength fiber does not satisfy the standard.

### 4.3. Correlation between Three-Point Bending Tests and Four-Point Bending Tests

The correlation model proposed by Conforti et al. [13] was adopted in Equations (4) and (5) to compare the test results obtained from the 3PBTs and 4PBTs. The measured displacements (CMOD and δ) obtained from the 3PBTs and 4PBTs were converted to CTOD values as normalized displacements. Similar tensile strength values were obtained for the same CTOD values in the 3PBTs and 4PBTs. The values of f_R1_, f_R2_, f_R3_, and f_R4_ from the 3PBTs are equivalent to the values of the residual strength at the deflections of 0.27 mm, 0.75 mm, 1.31 mm, and 1.85 mm obtained from the 4PBTs.

CTOD = 0.78 CMOD − 0.03(4)

CTOD = 1.5 δ − 0.05(5)

**Figure 13:**
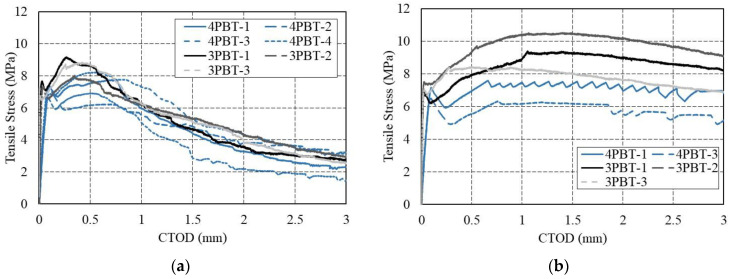
Relationship between tensile stress versus crack tip opening displacement (CTOD) obtained from results of three-point bending tests (3PBTs) and four-point bending tests (4PBTs): (**a**) 70-NF (normal-strength steel fiber) series and (**b**) 70-HF (high-strength steel fiber) series.

Figure 13a,b show the tensile stress versus CTOD curves obtained from the 3PBTs and 4PBTs for the specimens with normal-strength fiber (70-NF) and high-strength fiber (70-HF), respectively. Figure 13 indicates that the residual strength after peak loading obtained from the 3PBTs tends to be greater than that obtained from the 4PBTs. The differences in the specimens with high-strength steel fiber shown in Figure 13b are more noticeable than the differences in the specimens with normal-strength steel fiber shown in Figure 13a. Carrillo et al. [16] noted that such differences become more obvious as the fiber dosage and number of hooked ends increase. Therefore, these differences are a result of the tensile strength of the steel fiber.

**Figure 14:**
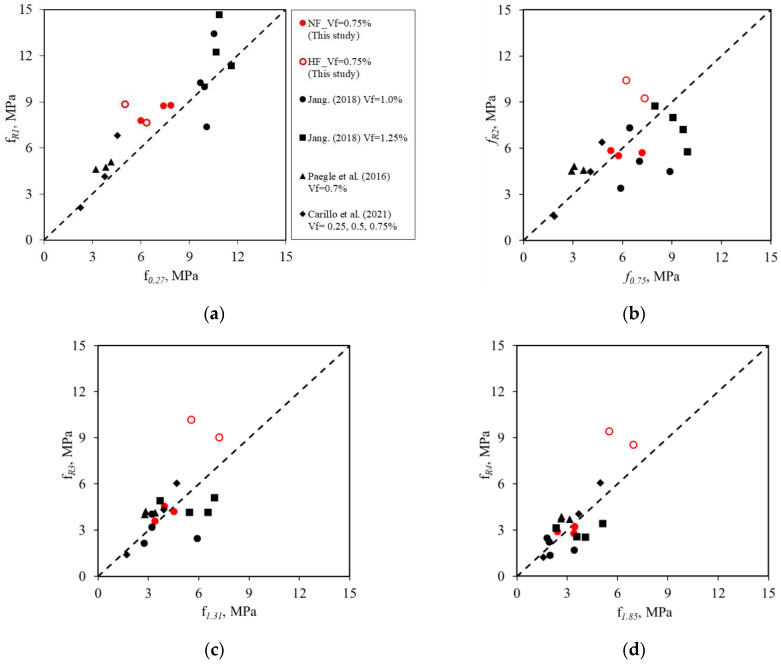
Comparison of the residual strength measured from 3PBTs and 4PBTs [9,16,31]: (**a**) *f_R_*_1_ vs. *f*_0.27_, (**b**) *f_R_*_2_ vs. *f*_0.75_, (**c**) *f_R_*_3_ vs. *f*_1.31_, and (**d**) *f_R_*_4_ vs. *f*_1.85_.

Figure 14 presents a comparison of the residual strength values measured from the 3PBTs and 4PBTs and includes test results from other studies that used similar tensile strength values (approximately 1200 MPa) for steel fiber. However, the compressive strength values of the SFRC differed in the various studies: 24 MPa, 57.1 MPa, 70 MPa, 80 MPa in Carrillo et al. [16], Paegle et al. [9], this study, and Jang [31], respectively. Figure 14a shows that the compressive strength of SFRC is the dominant influence on the residual strength in the early stages of testing. Figure 14b–d show that the tensile strength of the steel fiber affects the residual strength in the later stages. The residual strength values measured from the 3PBTs and 4PBTs are similar, regardless of the compressive strength of the SFRC, although the residual strength obtained from the 3PBTs is slightly greater than that obtained from the 4PBTs.

According to the analysis of variance conducted by Carrillo et al. [16], the parameters that lead to differences in the relationship between the 3PBT and 4PBT, such as the number of hooked ends, the reinforcement index, and the tensile strength of the steel fiber (*f_t_*), are independent variables in terms of determining the differences between the 3PBT and 4PBT due to the same compressive strength of the SFRC mixture. The adopted correlation model is applicable when using high-strength concrete with normal-strength steel fiber, but the correlation equations are limited for high-strength concrete with high-strength steel fiber.

## 5. Conclusions

Flexural performance of SFRC specimens was investigated in this study based on two standard specifications, EN-14651 (3PBTs) and ASTM C1609 (4PBTs), and two types of steel fiber, normal-strength and high-strength. Based on the experimental results, the following conclusions can be drawn.

The tensile strength of steel fiber embedded in high-strength concrete does not significantly affect the compressive strength and elastic modulus values of SFRC specimens.

The application of both standards, EN-14651 and ASTM C1609, led to compatible results that help provide a better understanding of the flexural performance of SFRC specimens. The use of EN-14651 for 3PBTs shows that the tensile strength of the steel fiber clearly affects the residual strength and energy absorption capacity during post-peak loading. Similarly, the use of ASTM C1609 for 4PBTs intuitively indicates that residual strength is influenced by the tensile strength of the steel fiber. However, an unintended cracking pattern outside the constant moment region was observed in 4PBT.

For the same volume fraction of steel fiber, the equivalent strength ratio and residual strength ratio tend to decrease. The criteria for ACI 318-19 and fib Model Code 2010 can be satisfied by increasing the tensile strength of the steel fiber rather than by increasing its volume fraction.

The correlation model shows that the flexural performance of SFRC is similar for 3PBTs and 4PBTs, but the residual strength obtained from the 3PBTs tends to be greater than that obtained from 4PBTs with an increase in the tensile strength of the embedded steel fiber.

## Figures and Tables

**Table 1:** Mixture proportions of steel fiber-reinforced concrete mixture used in this study.

W/B (%)	Air (%)	S/A (%)	Unit Weight (kg/m^3^)					
			W	C	SF	S	G	Steel Fiber
33	4	45	165	475	25	643	813	58.9

Note: W/B is water-to-binder ratio; S/A is sand-to-aggregate ratio; W is water; C is cement; SF is silica fume; S is sand; and G is gravel.

**Table 2:** Mechanical properties of steel fiber used in this study.

Type	Length	Diameter	Aspect Ratio	Tensile Strength
	(mm)	(mm)	(L/d)	(MPa)
Normal-strength steel fiber (NF)	35	0.55	64	1200
High-strength steel fiber (HF)	35	0.55	64	1600

**Table 3:** Compressive strength of steel fiber-reinforced concrete cylindrical specimens.

Mixture		*f_cu_* (MPa)	*E_c_* (GPa)	*ε_o_* (mm/mm)
70-NF	70-NF-1	67.4	32.9	0.0024
	70-NF-2	67.1	34.4	0.0024
	Average	67.2	33.6	0.0024
70-HF	70-HF-1	65.4	31.9	0.0025
	70-HF-2	62.9	31.3	0.0026
	70-HF-3	65.3	30.9	0.0027
	Average	64.5	31.3	0.0026

Note: *f_cu_* is compressive strength of SFRC; *E_c_* is elastic modulus; and *ε_o_* is ultimate strain.

**Table 4:** Effects of fiber tensile strength on flexural behavior of steel fiber-reinforced concrete (EN-14651 three-point beam tests).

Mixture	f*_L_* (MPa)	CMOD_F_*_L_* (mm)	f*_p_* (MPa)	CMOD*_p_* (mm)	f*_R_*_1_ (MPa)	f*_R_*_3_ (MPa)	f*_R_*_1_/f*_L_*	f*_R_*_3_/f*_R_*_1_
70-NF-1	7.67	0.040	9.17	0.34	8.72	3.61	1.14	0.41
70-NF-2	7.35	0.048	8.02	0.44	7.77	4.54	1.06	0.58
70-NF-3	7.26	0.011	8.84	0.54	8.77	4.21	1.21	0.48
Average	7.43 ± 0.18		8.68 ± 0.49	0.44	8.42 ± 0.46	4.12 ± 0.39	1.13	0.49
70-HF-1	6.93	0.029	9.40	1.35	7.63	9.01	1.10	1.18
70-HF-2	7.54	0.033	10.54	1.34	8.82	10.18	1.17	1.15
70-HF-3	7.11	0.039	8.45	0.66	8.31	7.69	1.17	0.93
Average	7.19 ± 0.26		9.46 ± 0.85	1.12	8.25 ± 0.49	8.96 ± 1.02	1.15	1.09

Note: 70-NF and 70-HF represent steel-fiber reinforced concrete mixtures with normal-strength steel fiber and high-strength steel fiber, respectively, at 70 MPa compressive strength; f_L_ is highest flexural strength in the interval of 0.05 mm.; CMOD_FL_ is CMOD at limit of proportionality (LOP); f_p_ is peak strength; CMOD_p_ is CMOD at peak strength; f_R_ is residual flexural strength.

**Table 5:** Effects of fiber tensile strength on flexural behavior of steel fiber-reinforced concrete (ASTM C 1609 four-point beam tests).

Mixture		f_1_ (MPa)	δ_1_ (mm)	f*_p_* (MPa)	δ*_p_* (mm)	R_600_	R_150_
70-NF	70-NF-1	7.38	0.06	7.78	0.50	1.04	0.31
	70-NF-2	7.32	0.07	7.32	0.07	0.84	0.44
	70-NF-3	6.70	0.05	8.24	0.38	1.13	0.45
	70-NF-4	6.61	0.06	6.92	0.34	0.94	0.23
	Average	7.00 ± 0.35	0.06 ± 0.01	7.56 ± 0.49	0.32 ± 0.16	0.99	0.36
70-HF	70-HF-1	7.13	0.06	7.60	0.44	1.03	0.98
	70-HF-2	6.50	0.06	6.50	0.06	0.97	0.79
	Average	6.86 ± 0.31	0.06 ± 0.00	7.05 ± 0.55	0.25 ± 0.19	1.00	0.89

Note: 70-NF and 70-HF represent steel fiber-reinforced concrete mixtures with normal-strength steel fiber and high-strength steel fiber, respectively, at 70 MPa; f_1_ is first-peak strength; δ_1_ is net deflection at first-peak load; f_p_ is peak strength; δ_p_ is net deflection at peak load; and R indicates equivalent flexural strength ratio.

## Data Availability

The data presented in this study are available on request from the corresponding author.
